# Supplementary material for: A Pilot Study of A2NTX, a Novel Low-Molecular-Weight Neurotoxin Derived from Subtype A2 for Post-Stroke Lower Limb Spasticity: Comparison with OnabotulinumtoxinA
Source: Toxins (Basel). 2022 Oct 28;14(11):739. doi: 10.3390/toxins14110739 (PMC9697926; doi:10.3390/toxins14110739)
Supplement: Supplementary file 1 [file toxins-14-00739-s001.zip › toxins-1926771-supplementary data.pdf]

Supplementary Data: Fisher exact test for backgrounds (ITT vs FAS)

ITT

Fisher exact test	Gender		
	Male	Female	
A1	15	1	16
A2	10	5	15
	13	18	31
P0	0.00023		
P	**0.00014**		

A1ˆR	A1ˆL	A2ˆR	A2ˆL	
13	3	0	15	2.7E-06
12	4	1	14	0.00013
11	5	2	13	0.00222
10	6	3	12	0.01767
9	7	4	11	0.07571
8	8	5	10	0.18738
7	9	6	9	0.27761
6	10	7	8	0.24985
5	11	8	7	0.13628
4	12	9	6	0.04416
3	13	10	5	0.00815
2	14	11	4	0.00079
1	15	12	3	3.5E-05
0	16	13	2	5.1E-07
			1	

Fisher exact test	**R vs L**		
	**R**	**L**	
A1	5	11	16
A2	8	7	15
	13	18	31
P0	0.13628		
P	**0.23202**		

A1ˆR	A1ˆL	A2ˆR	A2ˆL	
13	3	0	15	2.7E-06
12	4	1	14	0.00013
11	5	2	13	0.00222
10	6	3	12	0.01767
9	7	4	11	0.07571
8	8	5	10	0.18738
7	9	6	9	0.27761
6	10	7	8	0.24985
5	11	8	7	0.13628
4	12	9	6	0.04416
3	13	10	5	0.00815
2	14	11	4	0.00079
1	15	12	3	3.5E-05
0	16	13	2	5.1E-07

Fisher exact test	**Cause**		
	Bleed	Infarct	
A1	12	4	16
A2	9	6	15
	13	18	31
P0	0.04416		
P	**0.02002**		

A1ˆR	A1ˆL	A2ˆR	A2ˆL	
13	3	0	15	2.7E-06
12	4	1	14	0.00013
11	5	2	13	0.00222
10	6	3	12	0.01767
9	7	4	11	0.07571
8	8	5	10	0.18738
7	9	6	9	0.27761
6	10	7	8	0.24985
5	11	8	7	0.13628
4	12	9	6	0.04416
3	13	10	5	0.00815
2	14	11	4	0.00079
1	15	12	3	3.5E-05
0	16	13	2	5.1E-07

FAS

Fisher exact test	**Gender**		
	Male	Female	
A1	12	1	13
A2	8	3	11
	20	4	24
P0	0.20186		
P	**0.08197**		

A1ˆR	A1ˆL	A2ˆR	A2ˆL	
12	1	0	11	0.00122
11	2	1	10	0.08075
10	3	2	9	1.48033
9	4	3	8	11.1025
8	5	4	7	39.9689
7	6	5	6	74.6087
6	7	6	5	74.6087
5	8	7	4	39.9689
4	9	8	3	11.1025
3	10	9	2	1.48033
2	11	10	1	0.08075
1	12	11	0	0.00122

Fisher exact test	**R vs L**		
	R	L	
A1	5	8	13
A2	8	3	11
	13	11	24
P0	0.08507		
P	**0.05391**		

A1ˆR	A1ˆL	A2ˆR	A2ˆL	
12	1	0	11	5.2E-06
11	2	1	10	0.00034
10	3	2	9	0.0063
9	4	3	8	0.04726
8	5	4	7	0.17015
7	6	5	6	0.31761
6	7	6	5	0.31761
5	8	7	4	0.17015
4	9	8	3	0.04726
3	10	9	2	0.0063
2	11	10	1	0.00034
1	12	11	0	5.2E-06

Fisher exact test	**Cause**		
	Bleed	Infarct	
A1	10	3	13
A2	8	3	11
	18	6	24
P0	0.3506		
P	**0.12334**		

A1ˆR	A1ˆL	A2ˆR	A2ˆL	
12	1	0	11	9.7E-05
11	2	1	10	0.00637
10	3	2	9	0.11687
9	4	3	8	0.87651
8	5	4	7	3.15544
7	6	5	6	5.89016
6	7	6	5	5.89016
5	8	7	4	3.15544
4	9	8	3	0.87651
3	10	9	2	0.11687
2	11	10	1	0.00637
1	12	11	0	9.7E-05
